# Assessing perceived needs for telepathology implementation in Colombia: a baseline study from Red GLORIA

**DOI:** 10.3389/fdgth.2026.1765310

**Published:** 2026-05-12

**Authors:** Marcela Gomez-Suarez, Gabriel E. Acelas-Gonzalez, Andrés Mosquera-Zamudio, Julian C. Riaño-Moreno, Gabriela Guerron-Gomez, Maria Camila Restrepo-Guarnizo, Rafael Parra-Medina

**Affiliations:** 1 Instituto de Investigación, Universidad FUCS, Bogotá, Colombia; 2 Grupo INPAC, Laboratorio de Patología, Clínica Colsanitas, Bogotá, Colombia; 3 Universidad Cooperativa de Colombia, Villavicencio, Colombia; 4 Departamento de Patología, Instituto Nacional de Cancerología, Bogotá, Colombia

**Keywords:** cancer, oncology, pathologhy, public health, telepathology, latín America

## Abstract

**Background:**

Cancer diagnosis in Colombia faces delays and regional inequities, particularly in rural and underserved areas where access to subspecialist pathology is limited. General pathologists in these contexts frequently manage complex oncological cases with limited expert support, which may increase diagnostic uncertainty and professional burden.

**Methods:**

A concurrent mixed-methods design was applied within the GLORIA (Globalization of a telepathology network with artificial intelligence applications) project framework. Quantitative data were collected from a national survey distributed to ASOCOLPAT-registered pathologists (*n* = 57). Qualitative data were obtained through conversational interviews with general pathologists from six small and mid-sized laboratories (*n* = 11).

**Findings:**

Most participants reported needing subspecialist support for complex oncological cases (100%) and reported high emotional stress (86%), mainly associated with diagnostic uncertainty, legal responsibility, and excessive workload. Consultation response times differed across contexts, ranging from 1 to 7 days in Bogotá to more than 30 days in small cities. While 86% reported familiarity with telepathology, 63.2% had never used it in routine practice; however, 94% believed slide scanners could improve diagnostic timeliness. Qualitative findings identified recurrent themes related to administrative authorization barriers, logistical delays, professional isolation, and the perceived value of telepathology for timely expert feedback and learning.

**Interpretation:**

This baseline assessment suggests that telepathology implementation in Colombia must address not only technical readiness but also structural barriers in referral workflows and the professional needs of general pathologists, including timely feedback, shared responsibility, and learning opportunities.

## 1 Introduction

Cancer is officially classified as a high-cost disease by the Colombian government, according to the Colombian Fund for High-Cost Diseases. By 2020, the cancer burden among adults had reached a critical level, particularly affecting patients enrolled in the subsidized health insurance regime and those residing in remote or underserved areas. Among the major cancer types, cervical cancer had the longest average diagnostic waiting time (79.13 days), followed by prostate cancer (77.30 days) and breast cancer (70.25 days). Timely and high-quality histopathological evaluation is essential for accurate diagnosis and appropriate treatment planning (1). Despite this recognition, structural delays in pathology services and unequal access to subspecialty expertise continue to generate diagnostic inequities, particularly outside major urban centers. Addressing these inequities requires implementation-focused evidence to guide scalable telepathology solutions.

Over the past decade, Digital Pathology (DP) has seen significant advancement, particularly in high-income countries. This shift has enabled the integration of telepathology—a branch of telemedicine focused on pathology, which facilitates faster diagnosis, reduces geographic disparities in access to expertise, and promotes collaboration between general and specialized pathology laboratories across regions and countries (2–5). However, evidence describing baseline readiness, perceived needs, and contextual barriers to telepathology implementation in middle-income countries remains limited. Understanding these contextual conditions is essential for designing telepathology programs that are feasible and scalable in real-world health systems.

Although digital pathology has expanded globally, its implementation in Latin America—and particularly in Colombia—remains fragmented and insufficiently documented in real-world practice settings (6). In Colombia, access to DP technology remains limited, with only a few institutions currently equipped to use it (7). Its application has primarily been restricted to research contexts (8), rather than being adopted as a diagnostic tool for cancer and other pathologies (9). This technological gap presents a significant challenge, particularly for professionals working in general pathology laboratories who often manage complex diseases without access to specialized support. The national healthcare landscape reflects substantial disparities in access to oncological pathology services, hindering efforts to address the country's growing cancer burden. While healthcare coverage reaches approximately 95% of the population, timely access to care is significantly limited. This is because medical specialties are predominantly concentrated in the country's main cities, which make up just 3% of the national territory. This centralization creates notable inequalities, especially for populations in rural and intermediate areas. Moreover, the Colombian rate of pathologists per 100,000 inhabitants is 0.88, and there are no formal subspecialty training programs in oncological pathology in Colombia, leading to an uneven distribution of expert professionals. In this context, telepathology has been proposed as a strategy to support diagnostic consultation, reduce geographic barriers to specialist access, and strengthen professional collaboration across regions, although implementation feasibility depends on infrastructure, workflows, and sustained engagement of participating centers (10, 11).

To address these structural inequities, Project GLORIA (Globalization of a telepathology network with artificial intelligence applications) is a national DP initiative conceived in 2022 by a Colombian research team. Still under development, the project aims to reduce disparities in cancer diagnosis by connecting general pathology laboratories with specialized onychopathology centers through a shared DP network. It also seeks to create an image database to support the development of AI tools that enhance diagnostic accuracy and accessibility across the country (12).GLORIA is being developed as a collaborative telepathology network designed to connect pathology laboratories in small and medium-sized cities with specialized consultation centers. In practical terms, participating laboratories will be able to digitize selected complex cases and securely share whole-slide images with expert pathologists for review. The network is intended to support timely feedback, structured communication between requesting and consulting pathologists, and documentation of consultation outcomes.

The objective of this baseline phase was to identify perceived diagnostic challenges, implementation barriers, and readiness conditions among general pathologists working in small and medium-sized laboratories in Colombia. This baseline study was conducted prior to implementation to identify perceived needs, barriers, and readiness conditions that will inform workflow design, training priorities, and phased deployment across different regions in Colombia. In addition to enabling remote expert consultation, GLORIA includes a planned artificial intelligence (AI) component intended to support digital workflows, including case prioritization, quality control of scanned slides, and decision-support tools. By generating baseline implementation evidence, this study addresses the need for context-specific data to guide the operational design of a national telepathology network.

This study presents the evaluation of the first phase of the GLORIA project, developed within the RE-AIM framework (13) and grounded in implementation science approaches that support the translation of research into real-world practice. This phase represents a foundational step toward improving the timeliness and accuracy of cancer diagnosis and strengthening access to appropriate and timely treatment across Colombia (14).

## 2 Materials and methods

### 2.1 Study design

A concurrent mixed-methods approach within the RE-AIM framework was employed (15), with both qualitative and quantitative techniques conducted in parallel as described by Mosquera et al. (8). The RE-AIM framework provides a systematic approach for evaluating the public health impact of interventions. Its central objective is to direct attention to critical program components—particularly issues of external validity—that enhance the implementation of effective and generalizable strategies. By examining outcomes at both individual and organizational levels, the framework enables decision-makers to assess the extent to which interventions are successfully implemented in real-world contexts and to determine which warrant sustained investment.

**Figure 1:**
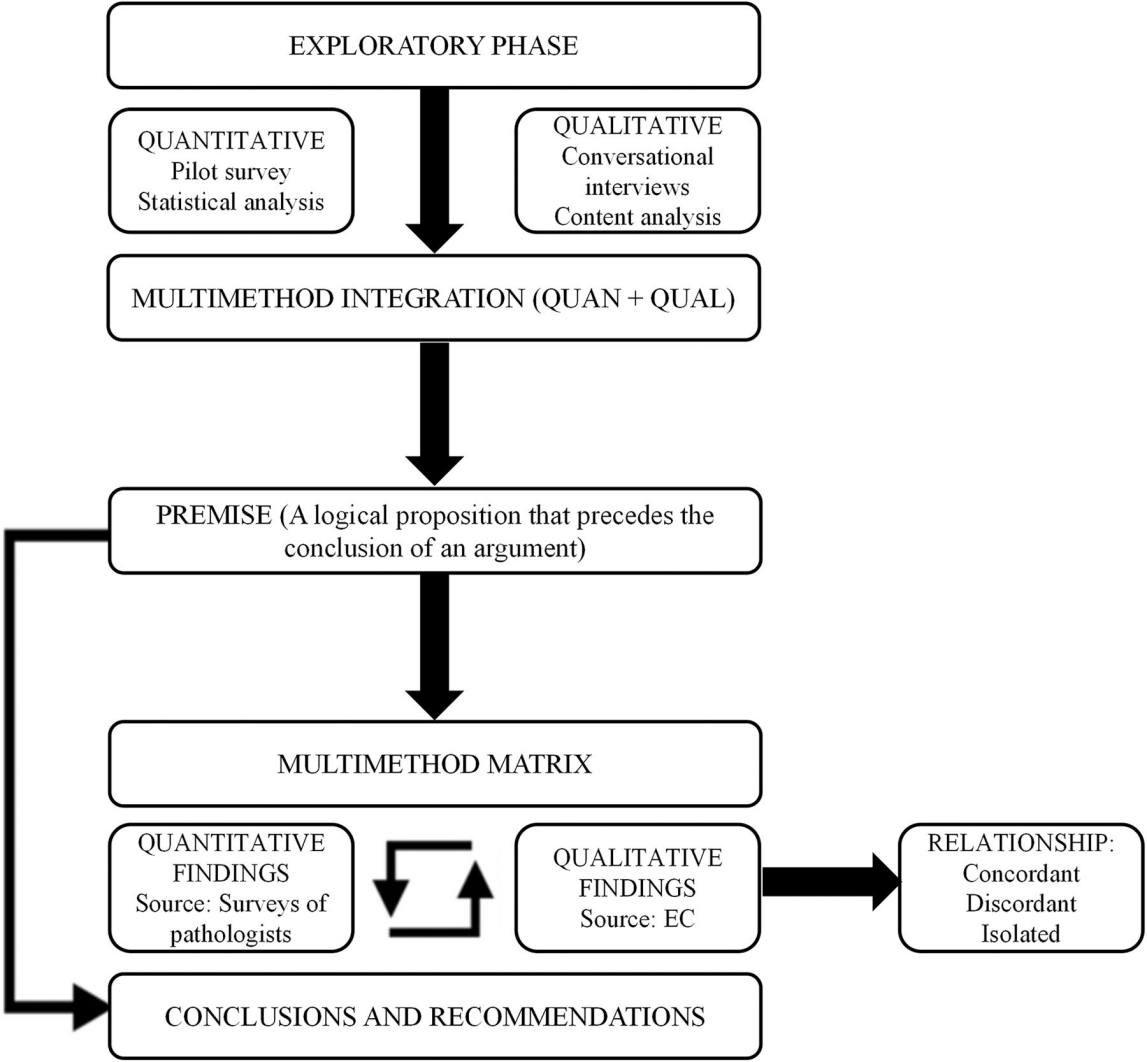
Multimethod analytical framework integrating quantitative and qualitative approaches; the figure presents the methodological design of a multimethod approach study structured in five main stages.

Using the first two components of the framework (Reach, effectiveness), this design allowed for a comprehensive analysis of baseline conditions in pathology laboratories, reaching the population of interest and capturing the subjective experiences of healthcare professionals alongside objective effectiveness on diagnostic timelines, workflows, and procedures (16). The process was structured into five distinct phases, which are illustrated in Figure 1: (1) exploratory phase, (2) multimethod integration, (3) development of logical premises, (4) construction of a multimethod matrix, and (5) reflective synthesis leading to conclusions and recommendations.

### 2.2 Recruitment and participating centers

The recruitment process targeted pathology laboratories from public and private hospitals, prioritizing institutions located in small and intermediate cities to capture regional diversity. Pathologists were invited through institutional agreements and professional networks, ensuring voluntary participation and representation across different levels of hospital complexity.

For the purposes of this study, small and medium-sized cities were defined according to population size and laboratory service capacity rather than cancer case volume. Small cities were defined as those with fewer than 1 million inhabitants and laboratories employing ≤2 practicing pathologists, while medium-sized cities were defined as those with 1–5 million inhabitants and laboratories employing 3–5 practicing pathologists. These operational definitions reflect diagnostic service capacity relevant to pathology consultation workflows.

At the time of this baseline study, participating centers were being considered as potential satellite digitization centers within the GLORIA implementation plan, which informed the assessment of feasibility, barriers, and contextual requirements for telepathology.

### 2.3 Exploratory phase

The exploratory phase was critical for understanding the context and baseline conditions of the participating laboratories. We conducted a contextual assessment in the regions where five small laboratories participating in the project are located, covering diverse areas of Colombia. Directors of the selected pathology laboratories shared detailed insights during interviews, describing routine processes such as sample reception, processing, diagnosis, and the referral of complex oncological cases.

### 2.4 Quantitative approach

We developed a pilot survey to examine how general pathologists' academic backgrounds and professional experience influence the diagnostic processes and timeliness of high-complexity tumor cases. Using a cultural and behavioral change (CBC) approach as a guide, the team structured the survey around three key categories: (1) diagnostic experience and timeliness, emphasizing the relationship between clinical experience and referral practices; (2) attitudes and emotional responses to complex oncological diagnoses; and (3) knowledge and readiness for telepathology.

Survey development followed a three-stage process: (1) item generation based on literature review and implementation-science constructs related to diagnostic workflow, workload, and technology readiness; (2) expert review by three senior pathologists and two implementation researchers to evaluate clarity, relevance, and content validity; and (3) pilot testing with five general pathologists not included in the final sample to assess comprehension and completion time, followed by minor wording adjustments prior to national distribution. These procedures ensured face and content validity of the survey instrument prior to national distribution.

The team designed and piloted a 25-item semi-structured questionnaire in Google Forms (Sup 1) and distributed it to ASOCOLPAT (Asociación Colombiana de Patología)-registered pathologists between March and June 2024. After performing data cleaning including validation, handling missing values, and consistency checks, we conducted statistical analyses using STATA version 15, applying descriptive statistics based on the type and distribution of each variable.

### 2.5 Qualitative approach

To develop the qualitative component, the research team conducted conversational interviews based on the phenomenological approach proposed by Van Manen (17), involving voluntary members of laboratory teams who had provided prior informed consent. Interviews followed a semi-structured conversational protocol designed to explore diagnostic workflow challenges, referral practices, professional experiences, and expectations regarding telepathology implementation. An interview guide ensured consistency across sites while allowing participants to elaborate on contextual experiences.

They carried out these interviews face-to-face at six pathology laboratories in different regions in Colombia before the implementation of the digital pathology solution and their designation as satellite digitization centers (or SDC) according to the GLORIA program, focusing on the challenges of diagnosing highly malignant tumors in laboratories without subspecialist pathologists. Participants shared narratives, anecdotes, and personal experiences during these sessions (17).

Each interview began with a phenomenological orienting question: *What challenges could be addressed by having a telepathology system in your laboratory?* This question encouraged participants to reflect on real situations, while follow-up discussions aimed to preserve the authenticity of their lived experiences. When necessary, interviewers use conversational techniques such as echoing responses or asking for specific examples to maintain the flow.

All interviews were digitally recorded using audio recorders and subsequently uploaded into NVivo 14 for systematic coding and analysis. Using a theoretical sampling model based on saturation, data collection continued until no new insights emerged. In the final stage, open coding was applied to develop conceptual categories that enriched, refined, or challenged the study's initial theoretical assumptions.

### 2.6 Multi-method integration

A multi-method integration strategy (QUAN + QUAL) (Figure 1) was employed within a concurrent mixed methods design. Guided by the framework of Tashakkori and Teddlie (18), a multimethod matrix was developed from an initial theoretical premise to systematically align the two components, enabling classification of findings as concordant, discordant, or isolated.

### 2.7 Ethical considerations

Participation in both the survey and interview components was voluntary. All participants were informed of their right to withdraw at any time. Informed consent was obtained before participation, outlining the study's objectives, voluntary involvement, confidentiality measures, and intended use of the collected data. The study was reviewed and approved by the Ethics Committee of the Fundación Universitaria de Ciencias de la Salud.

### 2.8 Next steps following the baseline phase

Based on the needs assessment reported in this manuscript, the next phase of GLORIA will focus on operationalizing a minimum digital pathology workflow for participating sites. This will include (1) defining criteria for case selection and referral (e.g., complex tumors requiring subspecialty input), (2) establishing secure procedures for whole-slide image acquisition and transfer, (3) identifying consultation centers with subspecialty expertise and defining response-time expectations, and (4) implementing training for laboratory staff and pathologists on slide scanning, data handling, and consultation communication. While the scale and sequencing of deployment will depend on institutional readiness and resource availability, these components will be necessary to transition from exploration to implementation and to evaluate feasibility under routine conditions.

## 3 Results

**Figure 2:**
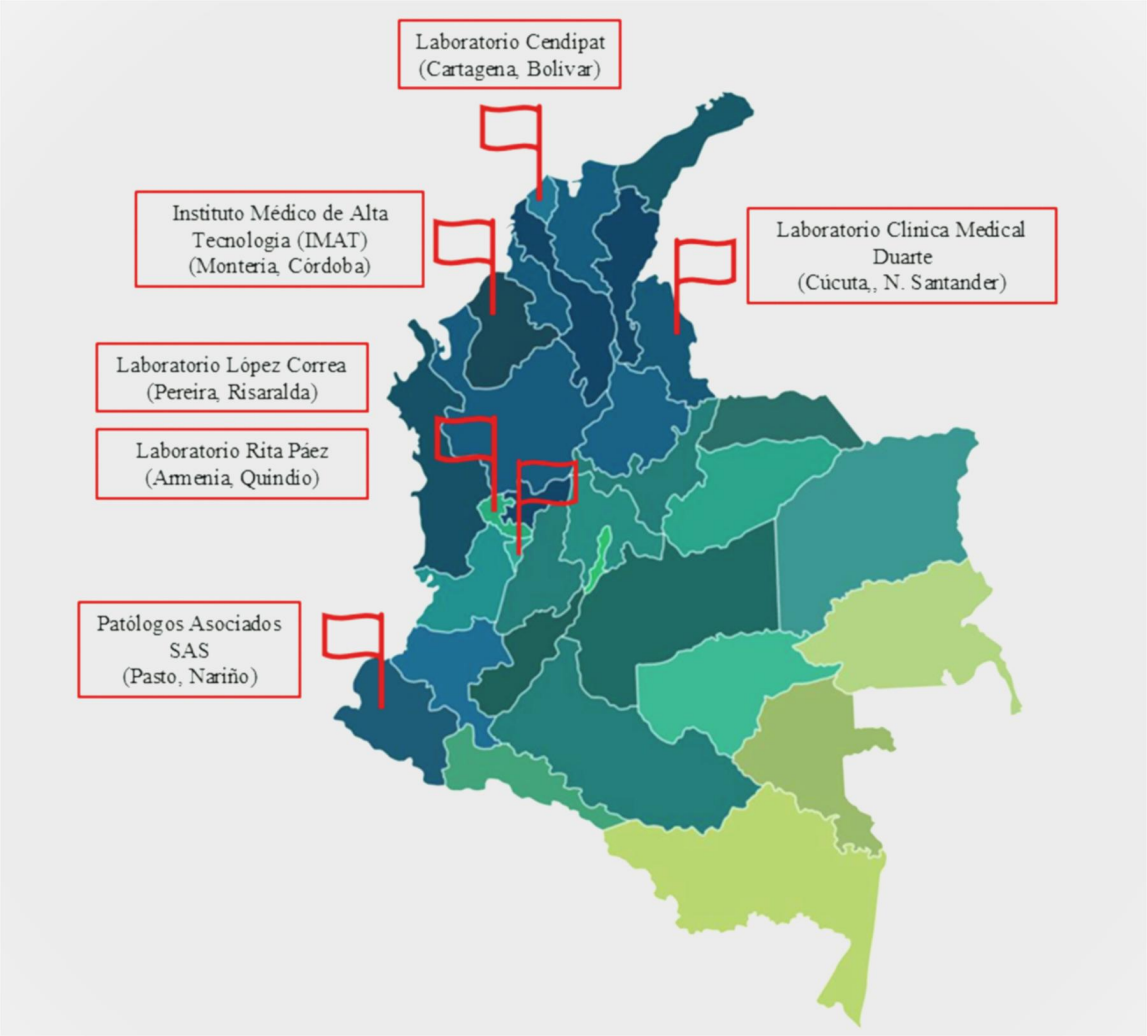
Geographic distribution of the regions and laboratories included in the study.

This section presents the main quantitative survey findings, followed by qualitative themes derived from interviews, and finally the integrated analysis of both components. The geographic distribution of the regions and laboratories assessed is presented in Figure 2 allowing a clearer understanding of the territorial scope and contextual diversity considered in this study.

### 3.1 Quantitative approach

A 25-question survey was distributed through the ASOCOLPAT network, yielding 57 responses. Bogotá (BOG) was analyzed separately due to its status as the capital and largest city. The remaining cities were grouped by population size into medium-sized (MED: 1–5 million) and small (SM: <1 million) categories.

Participants were predominantly women (65%), with a mean age of 49.3 years (SD = 12.3). Most respondents were general pathologists (*n* = 38), while 7 reported oncology subspecialty training and 6 reported other subspecialties. More than half had over 10 years of professional experience (*n* = 31).

Table 1 presents the basic characteristics of participating laboratories. Laboratory staffing differed substantially by region. Bogotá showed the highest concentration of larger laboratories (>5 pathologists), while MED and SM cities had a predominance of laboratories with smaller pathology teams (3–4 or 1–2 pathologists).

**Table 1:** Characteristics of participating pathologists.

Category	Variable	% (*n*) or Value
Demographics	Women	65%
	Average age (years)	49.3 (SD = 12.3)
Pathologists per City	BOG: >5/3–4/1–2	13/9/1
	MED: >5/3–4/1–2	2/6/3
	SM: >5/3–4/1–2	8/10/5
Training and Experience	General Pathologists	38
	Oncology Sub-specialists	7
	Other Sub-specialists	6
	>10 years of experience	31
Most Common Diagnoses	Digestive (stomach/intestines, esophagus)	96%/80%
	Respiratory (lungs, upper tract)	49%/47%
	Urinary (tract and bladder)	54%
Frequently Consulted Tumors	Melanocytic	69%
	Lymph nodes	63%
	Bone/Skin/Brain	53%/53%/50%
Support & Emotional Impact	Requires subspecialist support	100%
	Reports on high stress levels	86%
Response times to diagnostic consultations (days)	BOG	1–7
	MED	>7
	SM	>30

The table summarizes participants' demographic and professional profiles, including sex distribution, mean age, and the number of practicing pathologists per city. The data on pathologists per city reflect institutional diagnostic capacity, grouped by the number of pathologists per laboratory (>5, 3–4, or 1–2). Bogotá shows the highest concentration of large laboratories (13 sites with >5 pathologists), whereas MED and SM display a predominance of medium and small laboratories (6 and 10 sites with 3–4 pathologists, respectively). Additionally, levels of subspecialty training and experience. It also presents the most frequent diagnostic categories and tumor types consulted, the proportion requiring subspecialist support, and reported emotional stress levels. BOG: Bogotá. MED: Medium-sized city. SM: Small city.

All surveyed general pathologists (100%, *n* = 57) reported needing subspecialist support for complex oncology cases, and 86% reported high emotional stress associated with their diagnostic responsibilities. Consultation response times showed marked regional variation: respondents from Bogotá reported typical turnaround times of 1–7 days, while pathologists in medium-sized cities reported delays exceeding 7 days, and those in small cities frequently reported waiting periods longer than 30 days for subspecialist consultation. These results highlight substantial geographic disparities in access to timely diagnostic support.

**Figure 3:**
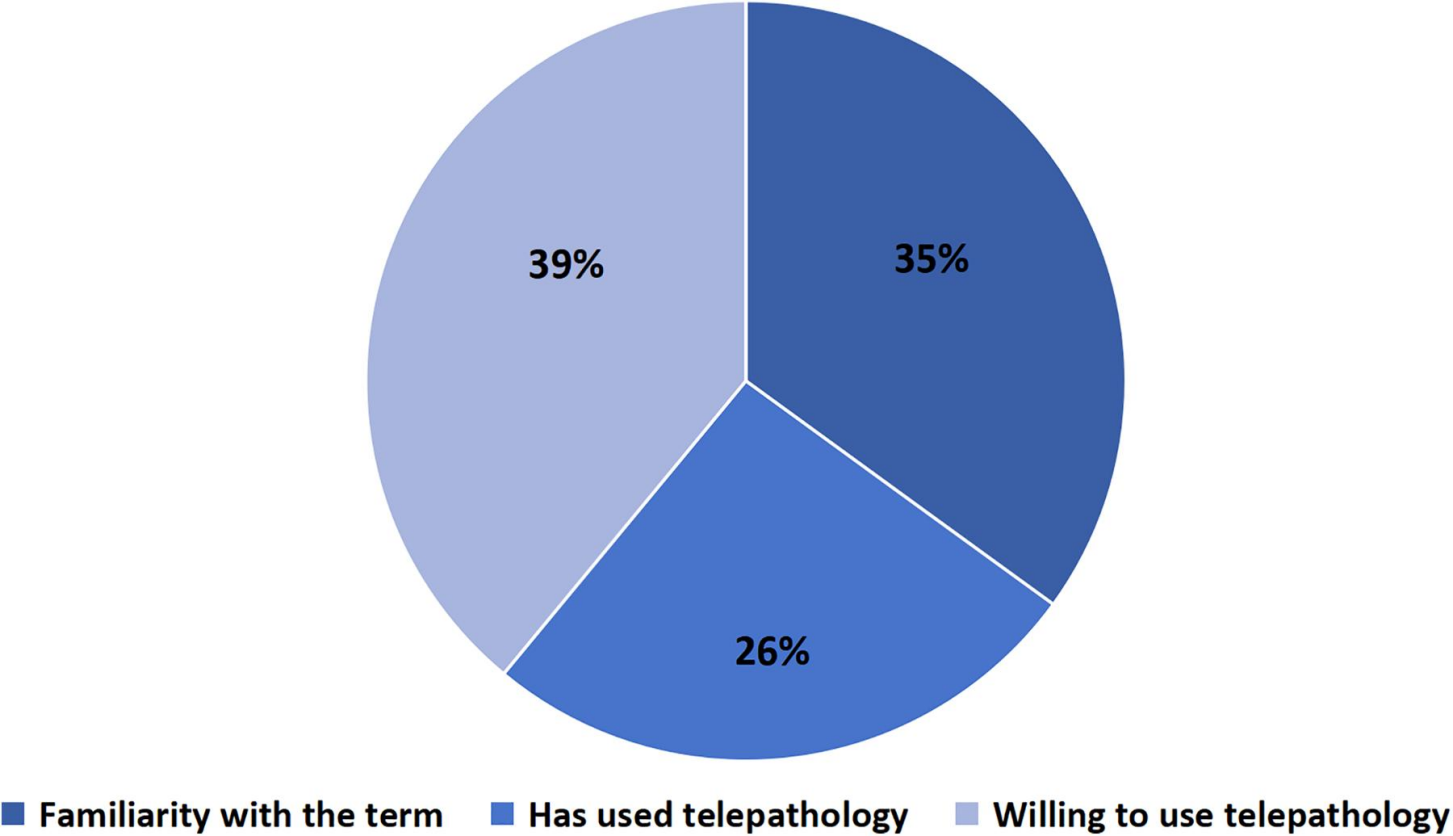
Distribution of responses regarding participants' experience and willingness to use telepathology.

Indicators of readiness for telepathology were relatively high despite limited practical experience. Although 86% of respondents reported familiarity with telepathology concepts, 63.2% indicated they had never used telepathology in routine practice. Nevertheless, 94% agreed that access to slide scanners could improve diagnostic timeliness, suggesting a favorable baseline perception toward digital pathology adoption (Figure 3). These findings suggest that, although actual use remains limited, there is a high level of openness and interest in adopting telepathology among participants.

### 3.2 Qualitative approach

As part of the qualitative component, 11 conversational interviews were conducted with general pathologists across six laboratories located in different regions of Colombia. The interviews explored participants' lived experiences and professional challenges related to cancer diagnosis in under-resourced settings, as well as their expectations regarding telepathology as a potential support strategy.

Thematic coding of the transcripts revealed six recurrent themes: (1) barriers in referral processes, (2) emotional stress, (3) professional isolation, (4) diagnostic collaboration practices, (5) learning needs and feedback expectations, and (6) anticipated benefits of telepathology for clinical efficiency and professional support.

**Figure 4:**
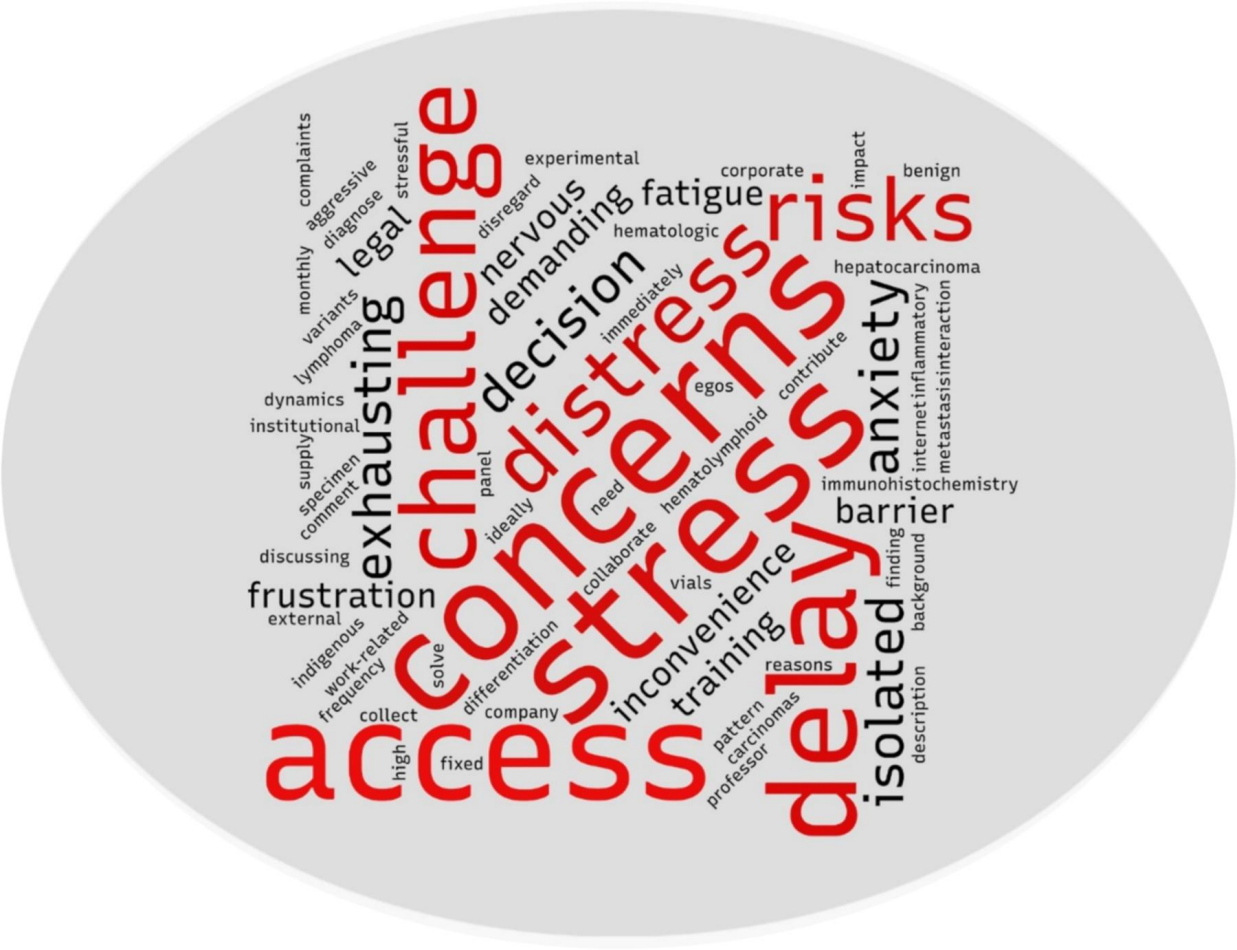
Word cloud representing the experiences of pathologists.

To complement the thematic analysis, we generated a word cloud (Figure 4) as a descriptive visualization of the most frequently occurring terms across interview transcripts. This figure is presented as a supportive aid to interpretation rather than a standalone analytic output. Prominent terms reflect recurring concepts related to referral delays, access constraints, emotional burden, and professional isolation described by participating pathologists.

The categories are presented in Table 2 and summarize the main experiences, opportunities, and challenges identified by pathologists during the interviews. Additional and expanded information is available in Supplement material S2.

**Table 2:** Qualitative findings.

Theme	Core Challenges	Illustrative Quote
Referral process	Delays, authorizations, logistics	“I can't send the sample without authorization…” (*Pereira 001*)
Emotional stress	Workload, fear of errors, legal risk	“It's stressful… you want to be precise.” (*Cúcuta 002*)
Professional isolation	Few colleagues, emotional strain	“I felt very alone… with so few people.” (*Monteria 003*)
Diagnostic collaboration	Consensus and responsibility	“It's better to refer the case. That's being responsible.” (*Monteria 003*)
Learning needs	Feedback and skills improvement	“You'd like to know how to approach certain cases…” (*Monteria 002*)
Telepathology expectations	Speed, peer learning, expert input	“It would greatly improve response times.” (*Cúcuta 002*)

Summary of qualitative findings organized by central themes, challenges identified and illustrative quotes from participating pathologists.

Recurring mentions of “delay” and “access” highlight the main barriers in referring complex cases, particularly in smaller laboratories. where authorization processes and logistics do not align with clinical urgency. As one participant explained: “*When prior authorization is needed, it gets delayed… some don't approve the referrals.”* (Armenia 001). Another participant linked this directly to access and reimbursement constraints: “*I can't send the sample without authorization because otherwise it won't be paid… it's a matter of access.”* (Pereira 001).

In addition to administrative barriers, participants described logistical difficulties in transporting samples or maintaining timely communication with colleagues across regions, especially in areas affected by geographic isolation or mobility disruptions: “*Traveling by road is very difficult… these delays and complicates interaction with colleagues.”* (Pasto 005). These barriers contributed to long waiting times for subspecialist review, often described as lasting weeks: “*In hematology… it takes more than 15 days.”* (Pereira 001) and “*Sometimes it takes between 20 days and a month.”* (Pasto 001).

Emotional “Stress” emerged as a central component across all narratives, often driven by high workloads, fear of error, and perceived legal risk. This was reflected in statements such as: “It's stressful… you want to be precise.” (Cúcuta 002) The emotional burden was heightened in life-altering cases, particularly among young patients: “*Not recognizing a tumor is extremely stressful …* *imagine, it could be a child who might die.”* (Montería 001).

General pathologists report that complex cases frequently lead them to seek second opinions from more experienced colleagues. This collaborative approach helps reduce individual responsibility and supports diagnostic decision-making. In some laboratories, peer discussion was framed as a practice that improves confidence and provides emotional relief: “*We've managed to hold pathology meetings… it's a shared responsibility that gives you peace of mind.”* (Cúcuta 003). When consensus was not possible, referral was described as a necessary and responsible step: “*If we all agree we can't reach a diagnosis, it’s better to refer the case. That’s being responsible.”* (Montería 003).

Professional isolation was particularly salient among participants working in smaller laboratories with limited peer support, reinforcing the emotional burden of high-stakes diagnostic decision-making. One interviewee stated: “I felt very alone… with so few people.” (Montería 003).

Participants also highlighted learning needs, emphasizing that consultation should not only resolve individual cases but also provide structured feedback that strengthens diagnostic skills over time. As one participant expressed: “You'd like to know how to approach certain cases…” (Montería 002).

Finally, telepathology was framed as a tool that could simultaneously improve efficiency and professional support. Participants expected faster expert input and improved collaboration, as illustrated by the statement: “It would greatly improve response times.” (Cúcuta 002).

### 3.3 Multi-method integration

Integration of quantitative and qualitative findings (QUAN + QUAL) enabled the formulation of logical premises based on baseline conditions, highlighting how structural constraints and professional experiences interact to shape diagnostic practices in general pathology laboratories. These premises constitute a theoretical basis for guiding future decisions on the implementation of diagnostic support strategies, such as telepathology, in contexts with structural limitations.

Across both components, findings converged in showing that workload and limited specialist availability contribute to diagnostic burden outside Bogotá (Table 3). Quantitatively, respondents described a high perceived need for subspecialist support (100%) and high emotional stress (86%). Qualitative narratives contextualized these responses by linking stress to high volumes, pressure to issue timely reports, and the consequences of uncertainty in high-stakes cases: “*It really is a bit stressful, because one would like to be more precise and not delay the pathology report.”* (Cúcuta 002) and “*Not recognizing a tumor is extremely stressful … imagine, it could be a child who might die.”* (Montería 001).

**Table 3:** Findings on workload, diagnostic complexity, delays, and stress.

Theme	Integrated Finding	Relationship
Workload and specialist distribution	Pathologists in Bogotá report better staffing and support; elsewhere, high volumes and complexity create overload.	Concordant
Diagnostic complexity	Labs in smaller cities regularly face 1–20 complex cases/month, many of which are oncological.	Concordant
Delays in diagnosis	In small/medium cities, over half report delays over a week; interviews confirm long waiting periods.	Concordant
Emotional burden and stress	High need for subspecialist support correlates with high stress, especially in PEQ and MED cities.	Concordant

Integrated qualitative and quantitative findings on workload, diagnostic complexity, delays, and stress among participating pathologists.

Integration also highlighted that diagnostic delays are not only related to clinical complexity but to system-level barriers. Survey results showed consultation response times differed by region (Bogotá: 1–7 days; medium-sized cities: >7 days; small cities: >30 days). Interviews confirmed that waiting periods of weeks are common in practice: “*In hematology… it takes more than 15 days.”* (Pereira 001) and *“Sometimes it takes between 20 days and a month.”* (Pasto 001).

Across both datasets, diagnostic collaboration emerged as an important coping strategy. Quantitative results showed that 82.5% of respondents reported preferring team-based diagnostic discussion before referring complex cases to subspecialists, reflecting an effort to reduce uncertainty and share responsibility in high-stakes cases. This pattern was repeated in interviews describing local case discussions as a source of reassurance: “*We've managed to hold pathology meetings … it's a shared responsibility that gives you peace of mind.”* (Cúcuta 003). At the same time, qualitative findings suggested that access to subspecialists may remain inconsistent, and communication is sometimes constrained by institutional processes. Participants described frustration when referrals depend on authorization or administrative approval, even in clinically urgent situations: “*When prior authorization is needed, it gets delayed… some don't approve the referrals.”* (Armenia 001) and “*I can't send the sample without authorization because otherwise it won't be paid … it's a matter of access.”* (Pereira 001). These barriers help explain why collaborative peer discussion often coexists with prolonged uncertainty and delayed specialist input.

Integration revealed high perceived readiness for telepathology despite limited practical experience (Table 5). While 86% of respondents reported familiarity with telepathology, 63.2% had never used it in routine practice; nevertheless, 94% believed that slide scanners could improve diagnostic timeliness. Interview narratives reinforced that telepathology is expected to reduce waiting times and improve support for both pathologists and patients: “*It would greatly improve response times. The main benefit would be huge for the patient.”* (Cúcuta 002).

Qualitative findings clarified that participants valued telepathology not only for faster expert input but also for learning and feedback that strengthens diagnostic skills over time: “*You'd like to know how to approach certain cases… and you learn from that.”* (Montería 002). Participants also emphasized that telepathology should function as guided support rather than replacing local clinical judgment: “*It would be great if the expert didn't say everything, but suggested: ‘Look, it might be this, send this immuno.’”* (Pasto 001).

Overall, the integrated findings presented in Tables 3–5 show a coherent baseline pattern: diagnostic complexity and delays contribute to stress and isolation, peer collaboration serves as a coping mechanism, and telepathology is perceived by participants as a potentially useful strategy to improve access to subspecialist consultation and reduce diagnostic delays.

**Table 4:** Findings on peer collaboration and communication barriers.

Theme	Integrated Insight	Relationship
Peer collaboration	Most general pathologists (82.5%) prefer team-based diagnosis before referring to specialists. Interviews affirm collaborative practices.	Concordant
Communication barriers	Despite the need for consultation, interviews reveal that specialists are often inaccessible, creating frustration and unresolved questions.	Isolated

Integrated qualitative and quantitative findings on peer collaboration and communication barriers in diagnostic practice.

**Table 5:** Findings on familiarity, readiness, and perceived benefits.

Theme	Integrated Insight	Relationship
Familiarity and readiness	While 86% are familiar with telepathology, 63.2% have never used it; interviews confirm low practical exposure.	Concordant
Perceived benefits	94% believe scanners could improve diagnostic timeliness; qualitative data reinforces its potential to benefit both pathologists and patients.	Concordant

Integrated qualitative and quantitative findings on familiarity, readiness, and perceived benefits of telepathology.

## 4 Discussion

The objective of this study was to establish a baseline assessment of diagnostic challenges, referral barriers, and perceived readiness for telepathology implementation among general pathologists in Colombia. This objective directly responds to the need for implementation-focused evidence identified in the Introduction. The findings provide implementation-oriented evidence indicating that diagnostic practices in general pathology laboratories are shaped by the interaction between structural constraints, professional experiences, and organizational workflows. Specifically, the observed delays in subspecialist consultation and the reported emotional burden suggest that diagnostic challenges are not solely related to clinical complexity, but to system-level limitations in access, coordination, and support.

Colombia, like many other middle-income countries, exhibits significant regional and cultural diversity. Geographic distances, limited infrastructure, and the fragmented delivery of healthcare services hinder residents of small cities from accessing the advanced cancer technologies available in major urban centers (19, 20). Consequently, most onco-pathologists choose to work in large cities, leaving general pathologists—often without specialized training—to handle complex oncological cases in smaller and mid-sized cities. These cases typically require inter-consultation, which delays final diagnoses three or four times more compared to large, well-equipped centers. Such delays contribute to severe inequities in cancer care and pose serious risks to patient outcomes and survival.

Similar challenges have been documented in other middle-income countries, where the centralization of cancer expertise in urban hubs leads to late diagnoses and poorer outcomes for rural and underserved populations (21). In Latin America, the lack of cancer specialists and uneven distribution of pathology services have been identified as critical barriers to early diagnosis and treatment, especially in countries like Peru, Bolivia, and Paraguay (22).

Our findings suggest that the barriers experienced by general pathologists are not limited to the absence of digital pathology tools. Instead, they reflect a combination of structural constraints—such as uneven distribution of specialist expertise, administrative referral processes, and geographic/logistical challenges—and professional factors, including emotional burden, isolation, and uncertainty in high-stakes diagnostic decision-making.

Quantitative results showed that all surveyed participants (100%) reported needing subspecialist support for complex oncological cases, and 86% reported high emotional stress associated with diagnostic responsibility. Consultation response times differed substantially across contexts, ranging from 1 to 7 days in Bogotá to delays exceeding 30 days in small cities. These differences suggest that access to timely expert input is influenced by regional resource distribution and organizational processes rather than clinical need alone.

Similar patterns of limited specialist availability and delayed interconsultation have been documented in pathology workforce studies performed in low and middle-Income countries, where structural deficits and resource constraints are linked to workload pressure and extended turnaround times (23).

Successful experiences have also been documented in countries such as India and Brazil, where national telepathology platforms have improved access to subspecialist input and reduced diagnostic turnaround times (24).

Qualitative findings help explain the mechanisms underlying these delays. Participants identified administrative authorization processes and reimbursement constraints as key factors that interrupt referral pathways, while logistical barriers—such as transportation difficulties and limited inter-institutional communication—further extend turnaround times. Together, these factors indicate that delays are embedded within workflow and governance structures rather than being isolated operational issues.

Challenges related to authorization and administrative processes align with broader telehealth implementation literature, which identifies governance, policy, and billing/authorization constraints as barriers to timely specialty access in low-resource settings (25).

High levels of reported stress (86%) appear to be closely associated with diagnostic uncertainty, workload, and the perceived consequences of error in high-stakes oncological cases. Qualitative narratives further indicate that this burden is intensified in settings with limited peer support, suggesting that professional isolation functions as a reinforcing factor in diagnostic stress.

At the same time, both datasets showed that peer-based collaboration is already used as a coping strategy: 82.5% of respondents reported team-based diagnosis before referral, and interviewees described local case discussions as a source of reassurance.

However, peer collaboration does not replace structured access to subspecialist input for rare or complex tumors, highlighting the importance of consultation models that support timely expert feedback and bidirectional communication.

Similar experiences have been reported in other resource-limited settings, where general pathologists face high diagnostic burdens without access to subspecialty support. A study in Nigeria and Ghana highlighted that pathologists frequently experience emotional strain, fear of error, and professional isolation when tasked with diagnosing complex cancer cases in the absence of timely expert input (26). These shared experiences underline the critical need for support networks and collaborative tools, such as telepathology, to mitigate the professional and emotional burden associated with high-stakes diagnostic decision-making.

The findings indicate a discrepancy between familiarity with telepathology (86%) and its routine use (63.2% had never used it), suggesting the presence of structural or operational barriers to adoption. Participants consistently identified telepathology as a potentially useful tool to improve access to subspecialist consultation and reduce delays. However, its implementation will depend on the extent to which identified barriers, particularly those related to infrastructure, training, and workflow integration are addressed.

Given the limited hands-on experience reported by many participants, implementation will likely require a minimum competency-based training package for both requesting and consulting pathologists, including basic scanning workflows, digital slide navigation, and standardized communication practices for interconsultation.

Studies in other contexts found that even in resource-constrained settings, most pathologists view telepathology favorably when accompanied by adequate infrastructure and training (3). Similarly, a study in Peru and Chile emphasized that successful telepathology implementation depends not only on hardware and connectivity, but also on the perceived value of peer interaction and the opportunity for professional learning (27). Recent surveys in pathology practice highlight this pattern of high awareness and optimism about digital pathology coupled with barriers to adoption, including cost, infrastructure, and training, which must be addressed to support sustainable implementation. Two 2025 surveys on pathologists' attitudes/knowledge toward digital pathology (Jordan and Brazil) documents high interest/awareness with practical gaps and highlights barriers like cost, infrastructure, training/feasibility (28, 29).

The RE-AIM framework used in this study is particularly valuable, as it provides a structured lens for anticipating the factors that will determine the success and sustainability of a telepathology network, anticipating implementation challenges across heterogeneous contexts. From this perspective, early “reach” and “effectiveness” considerations must be complemented by careful attention to adoption and implementation requirements, including technical infrastructure, training, workflow integration, and institutional readiness. These dimensions are particularly relevant in Colombia, where regional disparities in staffing and diagnostic capacity may influence the feasibility and sustainability of telepathology in real-world settings.

A critical implementation challenge is the financial model for subspecialty consultation services. While telepathology may reduce geographic barriers, expert review requires protected time and institutional support at consultation centers. In Colombia, sustainable implementation may require alignment with reimbursement mechanisms, either through public funding strategies, contractual agreements with insurers, or institutional cross-subsidization models. Within this context, Project GLORIA provides an implementation platform to evaluate whether telepathology can reduce consultation delays and support general pathologists in resource-constrained settings. Its effectiveness and sustainability will depend on the extent to which implementation processes address the structural and operational barriers identified in this baseline assessment. Future phases may evaluate diagnostic concordance, turnaround time, user experience, and sustainability under routine conditions before broader scale-up is considered.

This study has strengths, including its mixed-methods design, which enabled triangulation of quantitative patterns with qualitative lived experiences, and its inclusion of participants from diverse geographic settings. Limitations include the modest sample size, potential self-selection and self-report bias, and the fact that consultation response times and stress measures were reported by participants and may vary by institution and case type. Because participation was voluntary and the sample size was modest, findings should be interpreted as an implementation-oriented baseline assessment rather than a nationally representative estimate of all pathology laboratories in Colombia. Additionally, as a baseline study, these findings do not provide evidence of clinical impact or diagnostic accuracy improvements attributable to telepathology; these outcomes should be evaluated in subsequent phases of the project GLORIA.

Overall, this baseline study indicates that general pathologists in Colombia face substantial diagnostic burden, delays in accessing subspecialist input, and high emotional stress when managing complex oncological cases in under-resourced settings. Within the GLORIA implementation framework, telepathology is being explored as a strategy to address some of these structural and professional challenges. However, its effectiveness and sustainability will depend on the extent to which implementation processes successfully address administrative barriers, training needs, and workflow integration (26).

Project GLORIA provides an implementation context to evaluate whether telepathology can reduce consultation delays and support general pathologists in resource-constrained settings. Future phases should assess diagnostic concordance, turnaround time, user experience, and sustainability under routine conditions before broader scale-up is recommended.

Taken together, these findings suggest that telepathology implementation in Colombia should be understood as a system-level intervention rather than a purely technological solution. The interaction between infrastructure, administrative processes, and professional support mechanisms will likely determine whether digital pathology can effectively reduce diagnostic delays and improve working conditions in general pathology laboratories.

## 5 Conclusions

General pathologists in Colombia face high emotional stress, limited infrastructure, and institutional barriers that compromise timely cancer diagnosis. However, there is a strong willingness to integrate telepathology into routine practice. Project GLORIA's design must align with these realities to ensure successful and sustainable implementation. These findings have broader relevance for middle-income countries seeking to close diagnostic gaps through digital health innovation.

## Data Availability

The original contributions presented in the study are included in the article/Supplementary Material, further inquiries can be directed to the corresponding author.
