# Supplementary material for: Assessing perceived needs for telepathology implementation in Colombia: a baseline study from Red GLORIA
Source: Front Digit Health. 2026 May 12;8:1765310. doi: 10.3389/fdgth.2026.1765310 (PMC13201398; doi:10.3389/fdgth.2026.1765310)
Supplement: Supplementary file 1 [file Datasheet1.pdf]

# Supplementary Material

**Supplementary material 1 ......................................................................................1**
**Supplementary material 2 ......................................................................................7**

## Supplementary material 1

Structured survey administered to pathologists participating in the GLORIA Project. The instrument explores sociodemographic characteristics, laboratory infrastructure, diagnostic workload, frequency and type of interconsultations, perceived diagnostic challenges, and familiarity and attitudes toward telepathology.

**PROJECT GLORIA – Survey Form**

**SECTION 1: PERSONAL INFORMATION**

Please answer the following questions based on your personal data.

1. Full name*

2. Identification number*

3. How do you identify yourself?*
(Select one option)
☐ Male
☐ Female
☐ Other

4. Email address*

5. Age (in completed years)*

**SECTION 2: LABORATORY AND PROFESSIONAL INFORMATION**

Please answer the following questions based on the information related to the laboratory where you work and your professional background.

6. Name of the laboratory where you work*

7. City where the laboratory is located*

8. How many people work in your laboratory?
(Select all that apply according to the number of staff members)

| Role | 0 | 1–2 | 3–4 | 5 or more |
| --- | --- | --- | --- | --- |
| Pathologists | ☐ | ☐ | ☐ | ☐ |
| Cytotechnologists / Histotechnologists | ☐ | ☐ | ☐ | ☐ |
| Auxiliary staff | ☐ | ☐ | ☐ | ☐ |

9. What is your academic training level?*
(Select one option)
☐ General Pathologist → Skip to question 12
☐ Subspecialist Pathologist → Skip to question 13

10. How many years of experience do you have as a pathologist?*

11. How many years of experience do you have as a subspecialist?

12. What is your subspecialty?

**SECTION 3: CLINICAL EXPERIENCE**

Please answer the following questions based on your professional experience in the laboratory where you currently practice.

13. How many interconsultations do you perform per month?*

14. In your daily anatomic pathology practice, besides the surgical specimen, which of the following complementary data do you usually receive for diagnostic evaluation?
(Select all that apply)

| Data | Always | Sometimes | Never |
|---|---|---|---|
| Clinical history | ☐ | ☐ | ☐ |
| Imaging findings | ☐ | ☐ | ☐ |
| Surgical notes | ☐ | ☐ | ☐ |

Tissue types most frequently analyzed (indicate according to your experience)

For each system, please mark the degree of frequency that best describes your experience.

| System | Very frequent | Frequent | Occasionally | Rare |
|---|---|---|---|---|
| Central Nervous System (brain, spinal cord, peripheral nerves) | ☐ | ☐ | ☐ | ☐ |
| Respiratory System (lungs, upper airways) | ☐ | ☐ | ☐ | ☐ |
| Digestive System (esophagus, stomach, small and large intestines) | ☐ | ☐ | ☐ | ☐ |
| Urinary System (kidneys, urinary tract, bladder) | ☐ | ☐ | ☐ | ☐ |
| Reproductive System (testes, penis, ovaries, uterus, vagina) | ☐ | ☐ | ☐ | ☐ |
| Endocrine System (thyroid, parathyroid, adrenal glands) | ☐ | ☐ | ☐ | ☐ |
| Cardiovascular System (arteries, veins, capillaries) | ☐ | ☐ | ☐ | ☐ |
| Lymphatic System (lymph nodes, spleen) | ☐ | ☐ | ☐ | ☐ |
| Musculoskeletal System (bone, muscle, connective tissue) | ☐ | ☐ | ☐ | ☐ |

| System | Very frequent | Frequent | Occasionally | Rare |
|---|---|---|---|---|
| Skin and Appendages (melanocytic, non-melanocytic, adnexal tumors) | ☐ | ☐ | ☐ | ☐ |
| Head and Neck Region | ☐ | ☐ | ☐ | ☐ |

15. In your professional practice, have you ever felt the need for support from a subspecialist pathologist for high-complexity cases?*

(High-complexity cases are those presenting significant diagnostic challenges.)

☐ Yes

☐ No

16. Which types of tumors present the greatest diagnostic challenges for you?*

17. Considering those challenging tumors, what level of stress do you experience when issuing a diagnosis?*

☐ Very low

☐ Moderate but manageable

☐ High

☐ Uncontrollable

18. How many oncological cases per month do you encounter in which you experience diagnostic difficulties and decide (or wish) to seek an onco-pathologist's opinion?*

☐ 0

☐ 1–10

☐ 11–20

☐ 21–30

☐ 31–40

☐ 41–50

☐ 51–60

☐ 61–70

☐ 71–80

☐ 81–90

☐ 91–100

19. When you receive a high-complexity tumor for diagnosis, you usually…*

☐ Refer it immediately to an expert pathologist

☐ Attempt to diagnose it first, and refer only if necessary

☐ Do not receive high-complexity tumors

Tumor types most frequently referred for interconsultation (indicate frequency)

| System | Very frequent | Frequent | Occasionally | Rare |
|---|---|---|---|---|
| Nervous System | ☐ | ☐ | ☐ | ☐ |
| Respiratory System | ☐ | ☐ | ☐ | ☐ |
| Digestive System | ☐ | ☐ | ☐ | ☐ |
| Urinary System | ☐ | ☐ | ☐ | ☐ |
| Reproductive System | ☐ | ☐ | ☐ | ☐ |
| Endocrine System | ☐ | ☐ | ☐ | ☐ |
| Cardiovascular System | ☐ | ☐ | ☐ | ☐ |
| Lymphatic System | ☐ | ☐ | ☐ | ☐ |
| Musculoskeletal System | ☐ | ☐ | ☐ | ☐ |
| Skin and Appendages | ☐ | ☐ | ☐ | ☐ |
| Head and Neck Region | ☐ | ☐ | ☐ | ☐ |

20. How long does it usually take to receive an interconsultation report?*

☐ Between 1 day and 1 week

☐ Between 1 week and 1 month

☐ More than 1 month

**SECTION 4: KNOWLEDGE AND PERCEPTION OF TELEPATHOLOGY**

Please answer the following questions based on your current understanding of telepathology.

21. Are you familiar with the term “telepathology”?*

☐ Yes

☐ No

22. Have you ever used a telepathology service in your medical practice?*

☐ Yes

☐ No

23. How willing would you be to use a telepathology service in your workplace?*

☐ Very willing

☐ Willing, with prior training

☐ Slightly willing

☐ Unwilling

24. In your opinion, would a histological image scanner help improve diagnostic timeliness for high-complexity tumors?*

☐ Yes

☐ No

25. Additional comments or observations:

(You may use this space to share any relevant remarks.)

## Supplementary material 2

Expanded qualitative thematic matrix detailing the emerging categories, subcategories, specific challenges and representative quotes obtained during the interviews with pathologists.

| Key theme | Identified challenges | Representative quotes |
|---|---|---|
| **Experience related to the referral process for difficult diagnoses requiring interconsultation.** | | |
| **Delays due to authorizations** | The requirement of prior approval from insurance providers delays the referral and clinical decision making. | "When prior authorization is needed, it gets delayed… some don't approve the referrals." *(Armenia 001)* |
| **Administrative access barriers** | Inability to proceed without institutional approval, even when clinical urgency is evident. | "I can't send the sample without authorization because otherwise it won't be paid… it's a matter of access." *(Pereira 001)* |
| **Logistical difficulties** | Road or air transport is often complex, especially in regions with blockades or landslides. | "Traveling by road is very difficult… these delays and complicates interaction with colleagues." *(Pasto 005)* |
| **Long waiting times** | Specialist review and diagnosis can take between 10 days and a month, depending on the case. | "In hematology… it takes more than 15 days." *(Pereira 001)*"Sometimes it takes between 20 days and a month." *(Pasto 001)* |
| **Experiences related to anxiety, stressors, and fears** | | |
| **Workload and pressure** | High case volume, routine fatigue, pressure to deliver precise and timely reports. | "It really is a bit stressful, because one would like to be more precise and not delay the pathology report." (Cúcuta 002) |
| **Professional isolation** | Lack of long-term colleagues, feelings of being alone, especially in underserved areas. | "At the beginning I did feel very alone... managing that volume with so few people... it's been very difficult." (Monteria 003) |
| **Emotional burden of** | Fear of misdiagnosis in life-altering cases, especially in | "Not recognizing a tumor is extremely stressful... imagine, it could be a child who |

| | | |
|---|---|---|
| **diagnosis** | young patients. | might die.” (Monteria 001) |
| **Legal responsibility** | Risk of lawsuits or loss of professional credentials due to diagnostic errors. | “It’s always for legal reasons—that’s why we include: ‘oncologic pathology consultation requested.’” (Pereira 002) |
| **Experiences related to peer and subspecialist interaction processes** | | |
| **Diagnostic collaboration** | General pathologists work together to discuss difficult cases and reach a consensus. | “We’ve managed to hold pathology meetings… it’s a shared responsibility that gives you peace of mind.” (Cúcuta 003) |
| **Referral decision-making** | When consensus is not possible, referring to the case is seen as a responsible and necessary step. | “If we all agree we can’t reach a diagnosis, it’s better to refer the case. That’s being responsible.” (Montería 003) |
| **Experience related to the need for training and learning** | | |
| **Learning through interaction** | Pathologists want more than just answers — they seek feedback and guidance to strengthen skills. | “You’d like to know how to approach certain cases… and you learn from that.” (Monteria 002) |
| **Feedback as a learning tool** | Input from subspecialists is valued not only for resolving cases but for professional development. | “Someone who might know more and gives feedback — that would be a valuable learning opportunity.” (Cúcuta 002) |
| **Opportunities and expectations arising from the introduction of telepathology** | | |
| **Learning** | Enhance diagnostic skills through expert guidance. | “You gain experience and learn; your diagnostic level improves — it’s better for the patient.” *(Monteria 002)* |
| **Professional support** | Receive expert input without replacing individual clinical judgment. | “It would be great if the expert didn’t say everything, but suggested: ‘Look, it might be this, send this immuno.’” *(Pasto 001)* |
| **Peer interaction** | Exchange ideas and experiences among specialists. | “It’s about sharing experience, learning, and interacting.” *(Cúcuta 003)* |
| **Time** | Speed up diagnostic processes and directly | “It would greatly improve response times. The main benefit would be huge for the |

| | | |
|---|---|---|
| **optimization** | benefit patients. | patient.” *(Cúcuta 002)* |
